# Antioxidant Activities of Total Phenols of *Prunella vulgaris* L. *in Vitro* and in Tumor-bearing Mice

**DOI:** 10.3390/molecules15129145

**Published:** 2010-12-10

**Authors:** Liang Feng, Xiaobin Jia, Mao-Mao Zhu, Yan Chen, Feng Shi

**Affiliations:** 1 Biotechnology Laboratory of Chinese Medicine, Macau University of Science and Technology, Macau, China; E-Mail: wenmoxiushi@163.com (L.F.); 2 Key Laboratory of Delivery Systems of Chinese Meteria Medica, Jiangsu Provincial Academy of Chinese Medicine, Nanjing 210028, Jiangsu, China; E-Mails: ychen202@yahoo.com.cn (Y.C.); shifeng_1985_wcl@163.com (F.S.); 3 Analysis Center, Rudong County Grain Bureau, Nantong, 226400, Jiangsu, China; E-Mail: zhumaomao823@126.com (M-M.Z.)

**Keywords:** *Prunella vulgaris* L, total phenols, antioxidant activity, HPLC-DAD, SOD, MDA, tumor-bearing mice

## Abstract

*Prunella vulgaris* L. (PV, Labiatae) is known as a self-heal herb. The different extracts of dried spikes were studied for the best antioxidant active compounds. The 60% ethanol extract (P-60) showed strong antioxidant activity based on the results of 2,2’-azino-di(3-ethylbenzthiazoline-6-sulfonic acid (ABTS˙+), 2,2-diphenyl-1-picrylhydrazyl (DPPH) and ferric reducing antioxidant power (FRAP) assay methods. High performance liquid chromatography (HPLC) and LC/MS analysis showed that the main active compounds in P-60 were phenols, such as caffeic acid, rosmarinic acid, rutin and quercetin. Total phenols were highly correlated with the antioxidant activity (*R^2^* = 0.9988 in ABTS˙+; 0.6284 in DPPH and 0.9673 FRAP tests). P-60 could inhibit significantly the tumor growth in C57BL/6 mice. It can also been showed that increased superoxide dismutase (SOD) activity and decreased malondialdehyde (MDA) content in serum of tumor-bearing mice. These results suggested that P-60 extract had high antioxidant activity *in vitro* and *in vivo* and total phenols played an important role in antioxidant activity for inhibition of tumor growth.

## Abbreviations

(PV): *Prunella vulgaris* L.
(ABTS˙+): 2,2’-azino-di(3-ethylbenzthiazoline- 6-sulfonic acid
(DPPH): 2,2-diphenyl-1-picrylhydrazyl
(FRAP): ferric reducing antioxidant power
(SOD): superoxide dismutase
(MDA): malondialdehyde
(ROS): reactive oxygen species
(CTX): cyclophosphamide
(GSH): glutathione
(LPO): lipid peroxidation
(CAT): catalase
(HPLC): High performance liquid chromatography
(DAD): diode array detector
(SD): standard deviation

## 1. Introduction

Antioxidant effects play an important role in many human diseases, including cancer [1], diabetic complications [2], heart disease [3], liver damage [4], autism [5] and Alzheimer's disease [6], *etc*. Recently, reactive oxygen species (ROS) related to lipid peroxidation has been considered as one of the main causes of these diseases [7]. The protective effects of antioxidants on cell membrane lipid bilayers attacked by free radicals are attracting more interest. It has been reported that many compounds such as phenol acids, flavonoids, saponins, tannins, alkaloids and polysaccharides have antioxidant activity *in vitro* or *in vivo* [8,9,10]. These compounds are abundant in herbs and food additives. More recently, interest in the use of natural antioxidants from plants for the prevention and treatment of cancer has increased greatly [11], and oxidative stress was shown to influence treatment efficacy and survival of non-small cell lung cancer patients [12]. *Prunella vulgaris* L. (PV), with almost 15 known individual species widely distributed in Europe, Asia, northwestern Africa and North America, is known as a self-heal herb [13,14,35]. In China, PV has been used as an herbal medicine for thousands of years. It has been used to cure high blood pressure, headaches, lymphatic system disorder, goiter, tuberculosis, and tumors [15,16,17]. PV is found to exhibit significant antiestrogenic activity [18] and can induce apoptosis activity of tumor cells [19]. It is rich in phenolics, such as caffeic acid, rosmarinic acid, quercetin and rutin. These total phenolics show strong anti-tumor activity via different mechanisms [20,21,22,23,24]. It has been reported that *Prunella vulgaris* extract and rosmarinic acid can reduce reactive oxygen species production (ROS), intracellular glutathione (GSH) depletion as well as lipid peroxidation (LPO) [25]. However, according to our knowledge, there are a few reports about the use of PV extract as a natural antioxidant for inhibiting tumor growth via modulation of the SOD activity and MDA content. Therefore, the goals of the present study were to screen the PV extraction fraction showing antioxidant activity, to analyze the active antioxidant compounds and also investigate the antioxidant potential of PV extract on prevention of tumors *in vivo*.

## 2. Results and Discussion

### 2.1. Comparison on antioxidant activity of different PV extract fractions

The antioxidant activity of different PV extracts assayed by the ABTS, DPPH and FRAP methods [26,27,28,29,30,31,32]. As can be seen from Table 1, the TEAC, DPPH and FRAP values of P-60 were 89.307 μmol Trolox/g crude drug, 19.662 μmol Vc/g crude drug and 0.1567 μmol Fe(II)/g crude drug, respectively. The TEAC, DPPH and FRAP values of P-60 (extracted by 60% ethanol) were notable higher than those of the P-95 (extracted by 95% ethanol), P-30 (extracted by 30% ethanol) and P-w (extracted by distilled water) fractions. Moreover, the data from the ABTS, DPPH and FRAP tests was consistent, and indicated that P-60 fraction has the strongest potential antioxidant fraction and contains the most antioxidant compounds. 

**Table 1:** Comparison on antioxidant ability of different PV extracts by TEAC, DPPH and FRAP methods (n = 3).

Extracts	TEAC	DPPH value	FRAP value
	(μmol Trolox/g crude drug)	(μmol Vc/g crude drug)	(μmol Fe(II)/g crude drug)
P-95	8.926	13.710	0.0252
P-60	89.307	19.662	0.1567
P-30	37.335	17.645	0.0802
P-w	9.779	17.536	0.0558

### 2.2. HPLC analysis for antioxidant compounds

**Figure 1:**
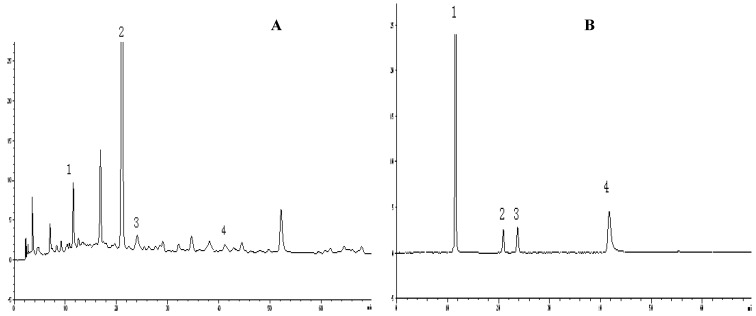
HPLC chromatograms of P-60 extract and the reference compounds.

**Figure 2:**
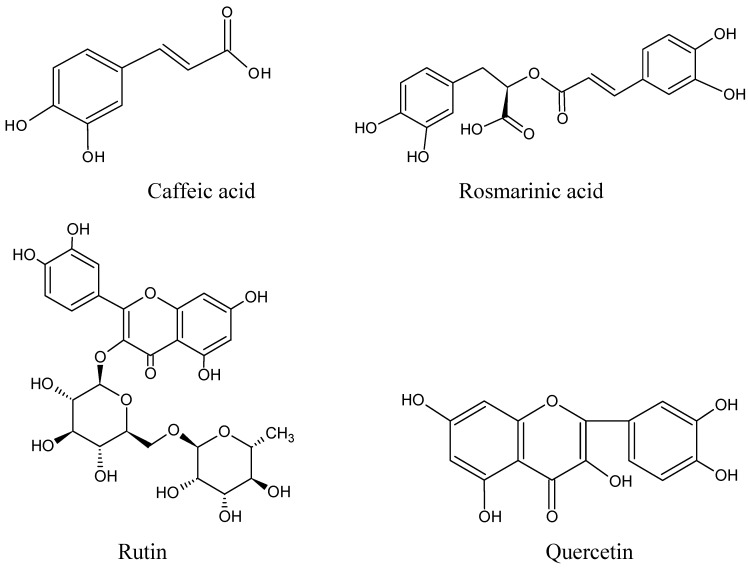
Chemical structures of major total phenol compounds in P-60.

Figure 1 shows HPLC chromatograms of the antioxidant compounds in P-60 and their reference standards. The results show caffeic acid (1), rosmarinic acid (2), rutin (3) and quercetin (4) were the main compounds in P-60. Chemically these are phenolic compounds (Figure 2). Of course, there are also some flavonoids in P-60, such as luteolin [33], but it also contains hydroxyl groups in the benzene ring, so therefore, it was also included in the total phenols. The LC/MS traces confirmed that the main compounds were caffeic acid, rosmarinic acid, rutin and quercetin, respectively.

The HPLC analysis indicated that while P-60 mainly contained phenolic compounds, the P-95 fraction mainly contain triterpenes and their saponins. This is because terpenoids are less polar than phenolic compounds, which are hydrophilic characteristic due to their hydroxyl groups.

### 2.3. The correlation between total phenols content and antioxidant activity

**Figure 3:**
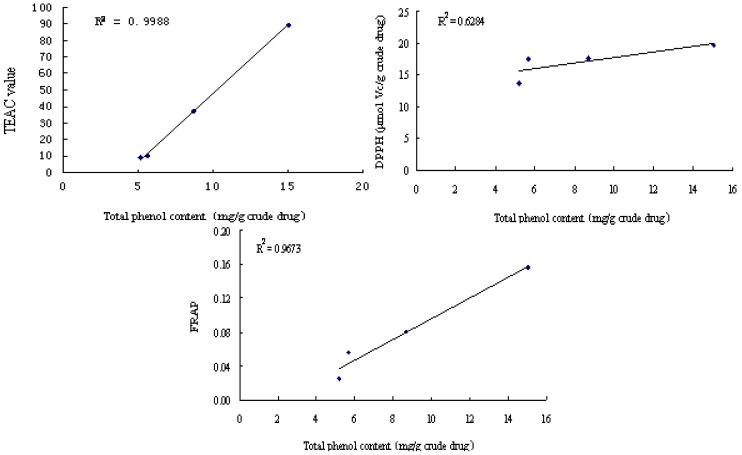
The correlation between total phenols content and TEAC, DPPH, FRAP values.

As can be seen in Figure 3, there is a high correlation between the total phenols content and antioxidant activity when the results from the TEAC, DPPH and FRAP tests are compared. The corresponding *R^2^* values were 0.9988, 0.6284 and 0.9608, respectively. These results showed that total phenols in P-60 were responsible for the antioxidant activity, so the P-60 fraction was selected for further study. 

### 2.4. Effect of P-60 on tumor growth in C57BL/6 mice

**Figure 4:**
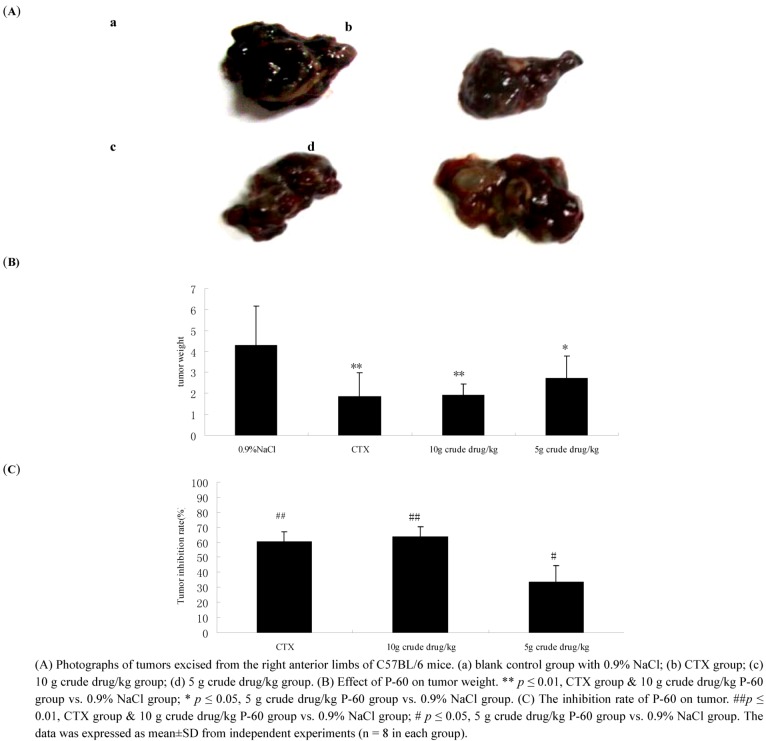
Efficacy of P-60 against tumor growth in C57BL/6 mice.

In Figure 4A, tumor volumes in the P-60 and cyclophosphamide (CTX) groups were visibly smaller than in the 0.9% NaCl group. The tumor weights of the 0.9% NaCl group, positive controlled CTX group (20 mg/kg), 10 g crude drug/kg P-60 group and 5 g crude drug/kg P-60 group were 4.27 ± 1.89, 1.84 ± 1.16, 1.90 ± 0.54 and 2.72 ± 1.06 g, respectively (Figure 4B). P-60 decreased significantly the tumor weight in P-60 treated group, compared with the 0.9% NaCl group (*P < 0.05), showing that P-60 had anti-tumor activity in *vivo*. As can be seen in Figure 4C, tumor inhibition rates (%) of the positive control, high and low dose of P-60 groups were 60.1 ± 6.8, 63.6 ± 6.79, 33.5 ± 1.10, respectively. There was a significant difference between the CTX and PV groups compared with the 0.9% NaCl group. (^#^ *P* < 0.05; ^##^ *p* < 0.01). 

### 2.5. Determination of total SOD activity and MDA content in tumor-bearing mice

**Figure 5:**
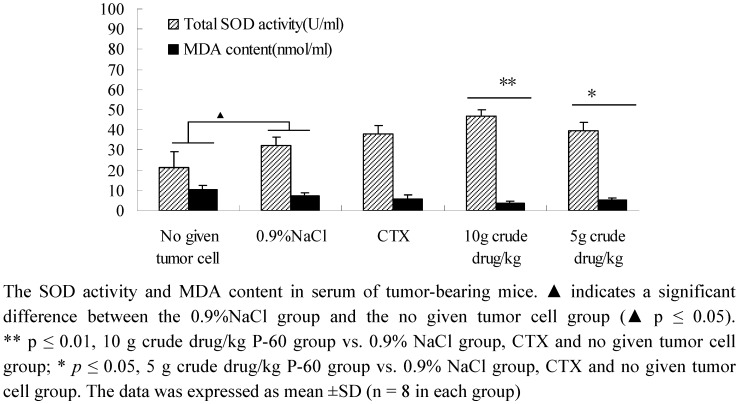
Effect of PV on SOD activity and MDA content in serum of tumor-bearing mice.

SOD is an organism-specific enzyme for scavenging free radicals. SOD activity in serum reveals the ability of the body and agents to remove oxygen free radicals. Malondialdehyde (MDA), the decomposition products of lipid peroxidation, reflects the severity of cell attack by free radicals. As can be seen from Figure 5, P-60 could significantly increase the SOD activity and decrease the MDA content in serum of tumor-bearing mice in a dose-dependent manner, compared with the untreated tumor cells and 0.9% NaCl groups. There were significant differences between the CTX group and P-60 treatment groups (*p* ≤ 0.01). To our surprise, SOD activity increased and the MDA content decreased in 0.9% NaCl group compared with the untreated tumor cell group, leading us to speculate that the balance of the *in vivo* oxidative stress system was disturbed. The above results indicated that P-60 had strong antioxidant activity in tumor-bearing mice and the antioxidants played an important role in tumor growth inhibition.

There are increasing reports that potential antioxidant of plants is related to their phenolic compound contents [34]. Phenolic compounds mainly include phenolic acids, flavonoids, saponins and tannins, all characterized by having hydroxyl groups in their benzene rings. It has been reported that phenolic compounds show antioxidant properties *in vivo* via modulation of glutathione (GSH) content, superoxide dismutase (SOD), catalase (CAT) activities, and malondialdehyde equivalent (MDA) [4]. Recently, it was reported that the antioxidant phenolic compounds in medical plants were highly correlated with the occurrence and development of tumors. The results indicate that the potential antioxidant activity of phenolic compounds may be related to modulating ROS caused by free radicals in tumors [33]. Therefore, phenolic compounds from medical plants can be used as antioxidant agents for preventing, reversing and delaying the occurrence and development of tumors. 

There are many active compounds in P-60, including mainly triterpenes like ursolic acid, oleanolic acid and its saponins; flavonoids like rutin, quercetin, luteolin; phenolic acids like caffeic acid, rosmarinic acid and its derivatives and polysaccharides [20,21,22]. Flavonoids and phenolic acids all contain hydroxyl groups that play a role in scavenging free radicals. Therefore, the flavonoids were included in the total phenols in this study. These phenolic compounds were metabolized *in vivo* via many bio-activating enzymes. After oral administration, rosmarinic acid, caffeic acid, and some metabolites such as dehydrogenase, caffeic acid, ferulic acid, and ferulic acid dehydrogenase could be detected in the serum, [34]. 

## 3. Experimental

### 3.1. General

UV-2802 UV-Vis spectrophotometer (UNIC, USA), Anke TGL-16G centrifuge (Shanghai Anke Scientific Instrument Factory), and an Agilent 1200 high performance liquid chromatography instrument (Agilent, USA) were used in this study. Methanol, acetic acid (TEDIA, USA) were chosen as mobile phase. Cyclophosphamide was ordered from Jiangsu Hengrui Medicine Co., Ltd., (Lianyungang, China, batch numbers: 08062524 and 08071721). SOD and MDA assay kits were ordered from Nanjing KeyGen Biotech. Co. Ltd. (Nanjing China, batch numbers: KGT001100, KGT7004, respectively). Dried spikes of PV were purchased from a local medicinal corporation of Bozhou, China. Herbs were authenticated as *Prunella vulgaris* L. by Professor D.K. Wu from the Nanjing University of Chinese Medicine.

### 3.2. Preparation of PV extract

Dried PV (4 kg) was refluxed successively in a 10-fold amount of 95% ethanol, 60% ethanol, 30% ethanol and water (2 times each, 2 h/run). The extract was concentrated by rotary evaporation at 60 °C and further dried in a vacuum oven at 60 °C. The crude yields obtained after extraction with 95% ethanol (P-95), 60% ethanol (P-60), 30% ethanol (P-30) and water (P-w) were 118.60 g, 179.98 g, 157.06 g and 156.96 g, respectively. 

### 3.3. HPLC analysis

The P-60 samples were analyzed on an Agilent 1200 high performance liquid chromatograph equipped with a quaternary pump, automatic sample injector, a diode array detector (DAD) and fitted with an Alltima C18 column (4.6 × 250 mm, 5 μm, USA). The column temperature was kept at 30 °C and samples were detected at 350 nm. The mobile phase was methanol and 0.1% glacial acetic acid. Samples were eluted with increasing methanol gradient as follows: 25 to 40% in 10 min, to 60% in 60 min, and held at 60% for 10 min The flow-rate was 1.0 mL/min and injection volume was 20 μL. The LC/MS system consisted of an Agilent 1200 series coupled to the TSQ Quantum mass spectrometer from ThermoFinnigan (San Jose, CA, USA). The ionization conditions under positive ESI were as follows: spray voltage: 4.5 kV; capillary temperature: 300 °C; collision pressure: 3.0 mTorr. The final sheath gas and auxiliary gas were set at 40 psi and 20 psi. Nitrogen was used as both nebulizing gas and auxiliary gas while Argon (Ar) was used as the collision gas.

### 3.4. Antioxidant activity by ABTS˙+, DPPH, FRAP in vitro

ABTS˙+, DPPH and FRAP methods were used to evaluate the antioxidant activity of the different PV extracts. A buffer system consisting of H_2_O_2_/ABTS/acetate was used for determining the ABTS˙+ clearance rate according to the method by Ozcan [26]. DPPH determination [26,27] and FRAP determination [28] were carried according to the indicated previously reported methods. Crude extracts of P-95, P-60, P-30 and P-w were analyzed by UV to study their antioxidation activity (equivalent to 1 g crude drug). The relationships between the total phenols in the mixture and the antioxidant activity of the extracts were then further investigated [29].

### 3.5. Effect of P-60 on tumor growth

Male C57BL/6 mice (18–20 g) of 6–8 weeks old were obtained from Shanghai Laboratory Animal Co., Ltd (SLAC). They were maintained under standard environmental conditions (temperature of 25 °C ± 2 °C and relative humidity of 50% ± 10%) and fed with a standard diet and water *ad libitum*. Primary tumors were induced in the right anterior limb by the subcutaneous (s.c.) injection of 0.2 mL cell suspension (106 Lewis cells). Mice in the no tumor cell group and the saline (0.9% NaCl) group were intragastrically administered 0.4 mL/day with 0.9% NaCl orally. Mice in the CTX control group were given 0.2 mL/day cyclophosphamide (20 mg/kg) by intraperitoneal injection. Mice in the P-60 group were given P-60 by intragastric administration orally (high dose: 10 g/kg/day, low-dose: 5 g/kg/day). After 14 consecutive days, the mice were weighed and sacrificed. The tumors were gently extracted and the tumor inhibition rate was calculated. Tissue samples were weighed and stored at −20 °C. Photographs of the tissue samples were taken using a sterecscope (SZX7, OLYMPUS, Canon camera) under auto-focus. 

### 3.6. SOD activity and MDA content in tumor-bearing C57BL/6 mice

Blood samples were taken from the orbital vein before the mice were sacrificed and centrifuged (4,000 rpm for 15 min) to segregate serum for the determination of SOD activity and MDA content. The serum samples were stored at −20 °C. The assays for SOD activity and MDA content in serum were performed according to the recommended methods provided in the kits.

### 3.7. Statistical analysis

All data were expressed as means ± standard deviation (SD), and analyzed by one-way ANOVA with the SPSS 16.0 software. Significant difference was investigated within and between the groups. Differences among means were determined with significance defined at *P* < 0.05.

## 4. Conclusions

In present study, P-60 extract of *P. vulgaris* L exhibited high free radical scavenging activity *in vitro* according to the ABTS, DPPH, FRAP methods. The main compounds in P-60 by HPLC analysis were phenolics which were highly correlated to the antioxidant activity. The growth of tumors was decreased and the SOD activity increased and the MDA content in serum decreased in tumor-bearing mice. The results indicated that the antioxidant effects of P-60 could play an important role in prevention and treatment of tumors, and we conclude that P-60 might be beneficial as a food additive or an anti-tumor agent.
